# Expanding the Mutational Landscape and Clinical Phenotype of CHD2-Related Encephalopathy

**DOI:** 10.1212/NXG.0000000000200168

**Published:** 2024-07-11

**Authors:** Angela Clara-Hwang, Stefani Stefani, Tracy Lau, Marcello Scala, Busra Aynekin, Pia Bernardo, Francesca Madia, Sophia Bakhtadze, Rauan Kaiyrzhanov, Reza Maroofian, Federico Zara, Varunvenkat M. Srinivasan, Vykuntaraju Gowda, Ulviyya Guliyeva, Alexandra Montavont, Anne-Lise Poulat, Ayten Güleç, Colette Berger, Dorothee M. Ville, Julitta de Bellescize, Sara Cabet, Antje Wonneberger, Alexander Schulz, Agusti Rodriguez-Palmero, Nicolas Chatron, Gaetan Lesca, Hüseyin Per, Himanshu Goel, Janis Brown, Tanja Frey, Katharina Steindl, Anita Rauch, Mariasavina Severino, Henry Houlden, Paola Nicolaides, Pasquale Striano, Stephanie Efthymiou

**Affiliations:** From the Department of Neuromuscular Disorders (A.C.-H., T.L., B.A., R.K., R.M., S.E., H.H.); Department of Clinical and Experimental Epilepsy (A.C.-H.), UCL Queen Square Institute of Neurology; The Francis Crick Institute (A.C.-H.), London, United Kingdom; Cyprus Paediatric Neurology Institute (S.S., P.N.), Nicosia, Cyprus; Department of Neurosciences, Rehabilitation, Ophthalmology, Genetics, Maternal and Child Health (M. Scala, P.S.), Università Degli Studi di Genova; U.O.C. Genetica Medica (M. Scala, F.Z.), IRCCS Istituto Giannina Gaslini, Genoa, Italy; Department of Neurosciences, Pediatric Psychiatry and Neurology (P.B.), Santobono-Pausilipon Children's Hospital, Naples, Italy; Medical Genetics Unit (F.M.), IRCCS Istituto Giannina Gaslini, Genoa, Italy; Department of Paediatric Neurology (S.B.), Tbilisi State Medical University, GA; Department of Pediatric Neurology (V.M.S., V.G.), Indira Gandhi Institute of Child Health, Bangalore, India; MediClub Hospital (U.G.), Baku, Azerbaijan; Department of Clinical and Functional Neurology (A.M., A.-L.P., C.B., D.M.V.), University Hospital of Lyon, Pierre-Bénite, France; Division of Pediatric Neurology (A.G., H.P.), Department of Pediatrics, Faculty of Medicine, Erciyes University, Kayseri, Turkey; Department of Paediatric Clinical Epileptology, Sleep Disorders and Functional Neurology (J. de Bellescize), University Hospitals of Lyon; Pediatric and Fetal Imaging Department (S.C.), Femme-Mere-Enfant Hospital, Hospices Civils de Lyon, Claude Bernard Lyon 1 University, France; Department of Neuropediatrics (A.W.), Jena University Hospital, Jena, Germany; MVZ Mitteldeutscher Praxisverbund Humangenetik GmbH (A.S.), Johannesstr. 147, Erfurt, Germany; Pediatric Neurology Unit (A.R.-P.), Pediatrics Department, Hospital Universitari Germans Trias I Pujol, Universitat Autonoma de Barcelona, Spain; Department of Genetics (N.C., G.L.), Hospices Civils de Lyon, France; NeuroMyoGene Institute (N.C., G.L.), CNRS UMR 5261-INSERM U1315, Claude Bernard Lyon 1 University, France; Hunter Genetics (H.G.), Waratah, NSW 2298, Australia; University of Newcastle, Callaghan, NSW 2308, Australia; John Hunter Children's Hospital (J. Brown), Australia; Institute of Medical Genetics (T.F., K.S., A.R.), University of Zurich, Zurich, Switzerland; (A.R.), University Children's Hospital Zurich; University of Zurich Research Priority Program ITINERARE: Innovative Therapies in Rare Diseases, AdaBD: Adaptive Brain Circuits in Development and Learning, Switzerland; Neuroradiology Unit (M. Severino.), IRCCS Giannina Gaslini Institute, Genoa, Italy; University of Nicosia Medical School (P.N.), Nicosia, Cyprus.

## Abstract

**Objectives:**

To present a case series of novel *CHD2* variants in patients presenting with genetic epileptic and developmental encephalopathy.

**Background:**

CHD2 gene encodes an ATP-dependent enzyme, chromodomain helicase DNA-binding protein 2, involved in chromatin remodeling. Pathogenic variants in CHD2 are linked to early-onset conditions such as developmental and epileptic encephalopathy, drug-resistant epilepsies, and neurodevelopmental disorders. Approximately 225 diagnosed patients from 28 countries exhibit various allelic variants in CHD2, including small intragenic deletions/insertions and missense, nonsense, and splice site variants.

**Results:**

We present the molecular and clinical characteristics of 17 unreported individuals from 17 families with novel pathogenic or likely pathogenic variants in *CHD2*. All individuals presented with severe global developmental delay, childhood-onset myoclonic epilepsy, and additional neuropsychiatric features, such as behavioral including autism, ADHD, and hyperactivity. Additional findings include abnormal reflexes, hypotonia and hypertonia, motor impairment, gastrointestinal problems, and kyphoscoliosis. Neuroimaging features included hippocampal signal alterations (4/10), with additional volume loss in 2 cases, inferior vermis hypoplasia (7/10), mild cerebellar atrophy (4/10), and cerebral atrophy (1/10).

**Discussion:**

Our study broadens the geographic scope of CHD2-related phenotypes, providing valuable insights into the prevalence and clinical characteristics of this genetic disorder in previously underrepresented populations.

## Introduction

Chromodomain helicase DNA-binding protein 2, CHD2, is a member of the ATP-dependent chromatin-remodeling proteins, involved in the assembly and regulation of chromatin.^1^ CHD2 and additional family members play an important role in chromatin structure remodeling, histone segregation, and deposition, which controls the 3D architecture of the genome and gene expression.^2^ The protein consists of 2 chromodomains, a putative helicase-binding ATP domain, and c-terminal domain..^3,4^ The ATP-helicase and DEDX-helicase domain are known to remodel the chromatin configuration by ATP-driven nucleosome remodeling complex driven.^4,5^

Heterozygous *CHD2* variants (MIM: 602119) have been identified in neurodevelopmental disorders characterized by early-onset epileptic encephalopathy and cognitive regression. Most patients experience multiple seizure types, including drop attacks, absences, myoclonic seizures, and photosensitivity associated with generalized spike-wave on EEG.^1^ Several prominent features of the CHD2‐myoclonic encephalopathy phenotype such as seizure type and sensitivity overlap with other developmental and epileptic encephalopathies (DEEs) including myoclonic‐atonic epilepsy, Lennox-Gastaut syndrome, West syndrome, and Jeavons syndrome.

We investigated 17 patients from 17 unrelated families carrying monoallelic *CHD2* variants who presented with childhood-onset myoclonic seizures, intellectual disability (ID), severe global developmental delay (GDD), and poor response to treatment. We expand on the genetic and phenotypic spectrum by reporting a novel recurring variant as well as motor impairments that were rarely reported in CHD2 studies.

## Methods

Our initial cohort comprised patients followed up in the Synaptopathies Patient Study Group (UCL), with additional families recruited through GeneMatcher. Monoallelic *CHD2* variants were identified by gene panel or exome sequencing (ES) and are listed using canonical transcript NM_001271.4. Allele frequency and pathogenicity of variants were assessed using available databases and in silico prediction software (eMethods).

### Standard Protocol Approvals, Registrations, and Patient Consents

The study was approved by the ethics IRB of UCL and additional local ethics committees of the participating research centers. Informed consent for the publication of clinical and genetic data was obtained from families.

### Data Availability

The corresponding author has full access to the data used in the analyses and takes full responsibility for the data, the analyses and interpretation, and the conduct of the research.

## Results

### Clinical Findings

We identified 17 patients from 17 families with monoallelic *CHD2* variants found by either trio or single ES (Figure, A) or panel sequencing (in family 2). The clinical features of the patients are summarized in eTable 1 and are shown in the Figure. Almost all individuals (except P13 and P16) presented with intractable early childhood-onset generalized epilepsy (mean 3.5 years, range 0.3–8.4 years), severe GDD, delayed speech, and ID. Three patients had microcephaly (P2, P4, and P13). Neurodevelopmental disorders affected more than half of the patients including autism spectrum disorder (ASD; 10/17, 59%) and attention-deficit hyperactivity disorder (ADHD; 11/17, 65%). Photosensitivity was also commonly observed in patients (11/17, 65%). Other variable features included status epilepticus (4/17, 24%), motor impairments (such as hypotonia (6/17, 35%) and hypertonia (3/17, 17%)), dysmorphic facial features (7/17, 41%), severe constipation (2/17, 12%), and kyphoscoliosis (3/17, 17%). Hyperopia was also observed in one patient (P1). Eight individuals (P1, P2, P4, P5, P12, P13, P14, and P17) had gross and fine motor skill difficulties.

**Figure:**
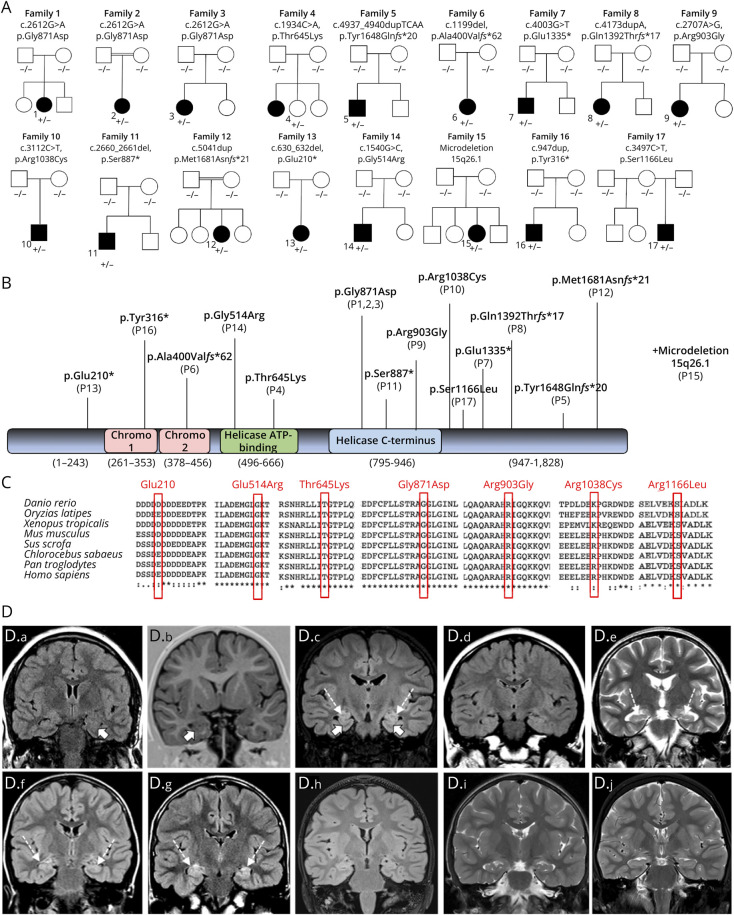
Pedigrees and Genetic and Radiologic Findings of the Patients (A) Pedigree of families 1–12. In the pedigree, squares represent men; circles represent women; black-shaded symbols denote affected patients harboring monoallelic CHD2 variants; and plus (+) and minus (−) signs indicate presence or absence of the CHD2 variants ([+/−] heterozygote and [−/−] wild type). (B) Schematic depiction of full-length CHD2 shows 2 chromodomains (pink), a helicase ATP-binding domain (green), and a helicase C-terminal domain (light blue). Variants identified in this cohort are displayed in bold and red. (C) The HomoloGene-generated amino acid alignment of human CHD2 and its predicted orthologs show the conservation of the amino acids Thr645, Gly871, Arg903, Arg1038, and Gly1575. (D) Brain MRI scans of Patient 1 performed at 13 years of age (a), Patient 2 at 3 years (b), Patient 4 at 8 years (c), Patient 6 at 7 years (d), Patient 7 at 11 years (e), Patient 8 at 7 years (f), Patient 11 at 11 years (g), Patient 14 at 5 years (h), Patient 15 at 4.5 years (i), and Patient 7 at 7 years of age (j). Coronal FLAIR or T2-weighted or IR images at the level of the hippocampi demonstrate unilateral or bilateral incomplete hippocampal rotation in Patients 1, 2, and 4 (thick arrows in a, b, and c). There is bilateral T2 or FLAIR hyperintensity of the hippocampi in Patients 4, 7, 8, and 11 (dashed arrows in c, e, f, and g). Additional mild hippocampal volume loss is noted in Patients 4 and 7.

### Neuroimaging Features

Brain MRI studies were available for review in 10 of 17 cases. The mean age at last MRI was 8 years (range 4.5–11 years). In 3 cases, follow-up MRI studies were performed with a mean follow-up duration of 5.5 years. Common findings were inferior vermis hypoplasia (7/10) and incomplete hippocampal rotation (3/10). Other observations included mild cerebellar subarachnoid space enlargement (4/10), periventricular white matter volume reduction (2/10), and focal signal alterations (1/10). In 4 of 10 cases, we identified bilateral T2/FLAIR hyperintensity of the hippocampi, associated with mild hippocampal volume loss in 2 patients. Longitudinal imaging showed stable hippocampal changes over 7 years in one case but mild cerebral and cerebellar atrophy progression in another over 1 year. MR spectroscopy studies were performed in 2 patients and were normal.

### Electroclinical Features of Epilepsy

All patients suffered from absences and eyelid myoclonia starting between 4 months and 8 years (mean 3.5 years). Light sensitivity with onset <3 years of life was common (9/14 patients, 64%), and occasional bilateral tonic-clonic seizures occurred in most individuals (9/14, 64%); convulsive or nonconvulsive status epilepticus was rare (3/14, 21%). Seizure frequency varied significantly, ranging from multiple episodes per day in some patients to experiencing seizures only every few months in others. Daily seizures were common in several patients, with variations in frequency and type, including myoclonic jerks, generalized tonic-clonic seizures, and absences. A few patients had less frequent episodes, such as one every 3 months or 5 episodes annually, indicating a broad spectrum of seizure severity and control within the group. EEG findings showed normal or mildly slowed background activity and interictal diffuse sharp wave/slow wave or polyspikes/slow wave complexes in most patients. Over the follow-up period, seizure management was challenging because of incomplete/poor response to multiple antiseizure medications (ASMs) in at least 50% of patients (P2, P6, P9, P11, P12). However, some individuals (P1, P4, P10, P13, P14) remained seizure-free on treatment for 5–8 years (eTable 2). Brain MRI was unremarkable except for 4 patients (P1, P4, P7 P11) showing nonspecific abnormalities, e.g., incomplete hippocampal rotation, deep collateral sulci, hypoplasia of inferior cingulum, and megacisterna magna (Figure, D).

### Genetic Findings

Among 15 variants identified, 10 variants were in the helicase C-terminal region of CHD2, except the p.Ala400Valfs*62 variant in the chromo-2 domain and p.Thr645Lys in the helicase ATP-binding domain (Figure, B). All variants had arisen de novo in the index probands, as confirmed by Sanger sequencing. Furthermore, missense variants occurred at highly conserved regions across species (Figure, C). Genetic information and allele frequency of each variant in external population databases are summarized in eTable 2.

In families 1, 2, and 3, a recurring novel heterozygous missense variant in exon 21, p.Gly871Asp, was identified, which has not been reported in publicly available population databases, as well as in more than 30,000 in-house exomes. A novel heterozygous nonsense variant, p.Glu1335* (family 7), is predicted to produce a premature translation termination, producing a truncated protein product that lacks the last 493 amino acids that contain part of the helicase C-terminal region. Similarly, the novel frameshift variants identified in families 11 (p.Ser887*) and 12 (p.Met1681Asnfs*21), both found in the adjacent C-terminal region, are absent from population databases. Another frameshift variant (p.Tyr316*) has been previously reported in VarSome. In family 8, a recurring variant, p.Gln1392Thrfs*17, is expected to result in an absent or disrupted protein product likely to undergo nonsense-mediated decay.

Novel heterozygous missense variants, p.Arg903Gly, p.Arg1038Cys, and p.Ser1166Leu, were identified in families 9, 10, and 11. The variant p.Arg1038Cys has been reported in one South Asian man in gnomAD. Both variants are predicted to have a deleterious impact on the CHD2 protein, affecting the same C-terminal catalytic stretch that contains the DNA-binding domain. In family 15, a 2.2-Mb deletion within the 15q26.1 region was identified by array analysis.

### Sex-Related Differences

The χ^2^ test for independence between sex and the occurrence of different genetic variants yielded a *p*-value of 0.233. Moreover, statistical analysis performed on the clinical features did not reveal any statistically significant differences between women and men (eMethods).

## Discussion

We report on the molecular and phenotypic spectrum of 17 individuals, with 11 novel variant sites and the first CHD2 pathogenic variants in Cypriot, Turkish, Georgian, and Azerbaijani populations, making the Eurasian region an important reservoir of genetic diversity, thereby an alluring site for conducting genetic studies.

While our patients are clinically consistent with known CHD2 cases, the spectrum of seizure types, treatment response, and motor symptoms were variable. In line with previous reports, almost all patients had infantile-onset or childhood-onset seizures although the seizure-onset age of P1 was 8 years 5 months, which deviated from previously reported data. Notably, 2 patients did not present with epilepsy, further supporting that epilepsy may not always be present in patients with CHD2 haploinsufficiency. It is noteworthy that motor dysfunctions were present in a small number of individuals in previous studies,^6^ yet our findings show that half of our patients, primarily those presenting with GDD, exhibited further neurologic features such as gait disturbances, abnormal reflexes, tremors, muscle tone, and/or fine motor dysfunctions. In addition, CHD2 was previously suggested as a genetic modifier for generalized photosensitive epilepsy syndrome, such as eyelid myoclonia with absences.^7,8^ Photosensitivity was reported in only 50% of patients while multiple seizure types with or without photosensitivity were observed in line with previous reports. CHD2's link to photosensitive epilepsy is acknowledged, but this study suggests that its impact extends beyond this condition alone. Photosensitivity is just one aspect among several variable features observed, requiring multiple EEG assessments for detection. However, the study lacks longitudinal EEG data across different developmental stages. Future studies should incorporate repeated EEG assessments to clarify relationship of CHD2 variants with photosensitivity and its evolution over time.

Neuroimaging features were generally nonspecific, including various findings like inferior cerebellar vermis hypoplasia, periventricular white matter volume loss, incomplete hippocampal rotation, mild cerebellar atrophy, and white matter gliotic changes.^9^ However, in 4 patients, a distinct hippocampal involvement pattern was noted, characterized by T2/FLAIR hyperintensity with mild volume reduction in 2 cases. This pattern remained stable over a 7-year follow-up in one patient, suggesting it may not progress to hippocampal sclerosis. While previous reports have noted hippocampal signal changes without accompanying volume loss, further investigation involving animal models and larger patient cohorts is warranted to understand their significance in CHD2 epileptic encephalopathy.^10^ Similar findings have also been documented in other epileptic disorders, such as Dravet syndrome^11^ and PCDH19 deficiency.^12^ Putative underlying mechanisms may involve Wallerian degeneration, apoptotic cell death, inflammation, and excitotoxicity.^13^

The *CHD2* variants identified were distributed throughout the protein but mainly focused on the helicase C-terminal region (871-1681aa). They are likely to have a comparable mechanistic effect, resulting in truncated CHD2 protein that lacks the C-terminus or affecting the region downstream of the DNA-binding domain, disrupting the C-terminus. A previously reported de novo variant (p.Gln1392Thrfs*17) also identified in our cohort (P8) further strengthens the potential mutational hotspot region that exists at the C-terminus.^6,14,15^

In addition to the variant being reported in ClinVar as likely pathogenic, the recurrent p.Gly871Asp variant represents a novel potential hotspot for pathogenic mutations found across the ethnic populations of Eurasia. Glycine at codon 871 is universally conserved across species and paralogs, implying a significant structural or functional role. All 3 patients had a history of failure to thrive, speech delay, developmental regression, DD, severe cognitive disability, ASD (P1, P2) or ADHD (P3), abnormal muscle tone, and gross motor impairments. Only P2 had poor response to ASMs and atonic seizures with eyelid myoclonia, and the first seizure onset ranged from 7 months to 8.5 years. Throughout the follow-up period, seizure management seemed challenging particularly in half of the patients who demonstrated resistance to multiple ASMs, indicating a complex refractory nature of their seizures. This underscores the necessity for continuous evaluation of therapeutic strategies, i.e., individualized and possibly multidisciplinary approaches to optimize seizure control.

Our cohort reports a significant proportion of individuals with neurodevelopmental disorders, i.e., ASD/ADHD with variable onset age, responsiveness to treatment, and symptom evolution, emphasizing the disease heterogeneity and the need for personalized management.

For clinical evolution, it is essential to note the variability among patients. For instance, P9, followed up for a long time, can offer valuable insights into the long-term disease progression, which can be crucial for developing targeted therapeutic strategies and providing appropriate care enhancing life quality.

Overall, our findings suggest that the same genotype can produce different clinical phenotypes in unrelated patients, with variable seizure onset, pharmacologic treatment response, photosensitivity, motor impairment, and behavioral or cognitive skills.

Notably, children born to consanguineous parents in families 2 and 12 have been diagnosed with de novo variants, underlying the necessity of evaluating dominant inheritance in ES data. De novo variants are often overlooked in consanguineous families, where homozygous variations are the usual suspects. Despite that, consanguinity in family 2 could account for the dissimilar phenotype severity compared with families 1 and 3, in addition to the *CHD2* variant being identified by epileptic encephalopathy panel testing instead of ES.

Recent studies have revealed sex-specific differences in both electroclinical features and prognostic factors in patients with genetic generalized epilepsy.^16^ In this series, there was no significant difference in the distribution of variants and clinical features between men and women.

Although CHD2-related epilepsy is clinically well established, the molecular and functional mechanisms of CHD2 are continuously being investigated. De novo loss-of-function pathogenic variants have been associated with clinical and electrographic seizures in animal models resembling those presented by patients. CHD2 knockdown in zebrafish disturbed locomotor activities and caused epileptiform discharges.^17^ EEG studies in CHD2-knockdown mice revealed elevated resting alpha and gamma frequencies as well as increased cortical synchrony in humans.^18^ Our patients displayed generalized seizure types and ictal/interictal slow wave complexes despite epilepsy severity, treatment, and independent of stimulus, supporting the intrinsic dysfunction of the epileptic network resulting in an overall increased cortical synchronicity.^19^

CHD2-related epilepsy often manifests with diverse seizure types alongside developmental disabilities, autism, and photosensitivity. Our EEG findings are consistent with previous literature, showing intractable seizures necessitating multiple antiseizure medications and EEG patterns of normal or mildly slowed background with sharp/slow wave complexes. These results support the link between CHD2 and a spectrum of neurodevelopmental conditions.

Some significant phenotypes across age groups are as follows: in infancy (0–2 years), early-onset seizures and developmental delays, such as motor delay, are prominent; during early childhood (3–5 years), there are neurodevelopmental challenges like language delays or social interaction difficulties, often alongside more distinct seizure types; school-age children (6–12 years) may experience academic struggles, learning disabilities, or ADHD; adolescence (13–18 years) brings about behavioral issues like hyperactivity, ASD, and potential shifts in seizure patterns; and in adulthood (18+ years), seizures tend to persist alongside ID and psychiatric symptoms.

Phenotypic variability observed in individuals with *CHD2* might be accounted for in several ways. First, the gene is ubiquitously expressed in all human tissue. CHD2 expression levels change across different regions of the neocortex during embryonic development in animal models.^6^ Second, CHD2, as a chromatin remodeler, alters the expression of developmental transcriptional regulators, e.g., the repressor element 1-silencing transcription factor (REST) gene, a key regulator of epileptogenesis.^20^ Finally, during the DNA repair process, CHD family proteins interact and comodify tissues, compensating for haploinsufficiency.^2^

In conclusion, we describe 17 novel patients with 11 novel de novo pathogenic *CHD2* variants, expanding both the genotypic and mutational phenotypic spectrum of the disorder. We report a potential novel hotspot mutation in the important helicase C-terminal region. We show that individuals with *CHD2* variants can present with gross and fine motor dysfunction and they do not always present with photosensitive epilepsy syndromes.

## Appendix Authors

**Table:**

Name	Location	Contribution
Angela Clara-Hwang, MSc	Department of Neuromuscular Disorders; Department of Clinical and Experimental Epilepsy, UCL Queen Square Institute of Neurology; The Francis Crick Institue, London, United Kingdom	Drafting/revision of the manuscript for content, including medical writing for content; major role in the acquisition of data; study concept or design; analysis or interpretation of data
Stefani Stefani, MSc	Cyprus Paediatric Neurology Institute, Nicosia, Cyprus	Major role in the acquisition of data; analysis or interpretation of data
Tracy Lau, MSc	Department of Neuromuscular Disorders, UCL Queen Square Institute of Neurology, UCL, London, United Kingdom	Major role in the acquisition of data; analysis or interpretation of data
Marcello Scala, MD	Department of Neurosciences, Rehabilitation, Ophthalmology, Genetics, Maternal and Child Health, Università Degli Studi di Genova; U.O.C. Genetica Medica, IRCCS Istituto Giannina Gaslini, Genova, Italy	Major role in the acquisition of data; analysis or interpretation of data
Busra Aynekin, PhD	Department of Neuromuscular Disorders, UCL Queen Square Institute of Neurology, UCL, London, United Kingdom	Drafting/revision of the manuscript for content, including medical writing for content; major role in the acquisition of data; analysis or interpretation of data
Pia Bernardo, PhD	Department of Neurosciences, Pediatric Psychiatry and Neurology, Santobono-Pausilipon Children's Hospital, Naples, Italy	Major role in the acquisition of data; analysis or interpretation of data
Francesca Madia, PhD	Medical Genetics Unit, IRCCS Istituto Giannina Gaslini, Genoa, Italy	Major role in the acquisition of data; analysis or interpretation of data
Sophia Bakhtadze, PhD	Department of Paediatric Neurology, Tbilisi State Medical University, GA	Major role in the acquisition of data; analysis or interpretation of data
Rauan Kaiyrzhanov, MD, PhD	Department of Neuromuscular Disorders, UCL Queen Square Institute of Neurology, UCL, London, United Kingdom	Major role in the acquisition of data; analysis or interpretation of data
Reza Maroofian, PhD	Department of Neuromuscular Disorders, UCL Queen Square Institute of Neurology, UCL, London, United Kingdom	Major role in the acquisition of data; analysis or interpretation of data
Federico Zara, PhD	U.O.C. Genetica Medica, IRCCS Istituto Giannina Gaslini, Genova, Italy	Major role in the acquisition of data; analysis or interpretation of data
Varunvenkat M. Srinivasan, MD, DM	Department of Pediatric Neurology, Indira Gandhi Institute of Child Health, Bangalore, India	Major role in the acquisition of data; analysis or interpretation of data
Vykuntaraju Gowda, MBBS, PhD	Department of Pediatric Neurology, Indira Gandhi Institute of Child Health, Bangalore, India	Drafting/revision of the manuscript for content, including medical writing for content; major role in the acquisition of data
Ulviyya Guliyeva, MD, PhD	MediClub Hospital, Baku, Azerbaijan	Major role in the acquisition of data; analysis or interpretation of data
Alexandra Montavont, MD	Department of Clinical and Functional Neurology, University Hospital of Lyon, Pierre-Bénite, France	Major role in the acquisition of data; analysis or interpretation of data
Anne-Lise Poulat, MD	Department of Clinical and Functional Neurology, University Hospital of Lyon, Pierre-Bénite, France	Major role in the acquisition of data; analysis or interpretation of data
Ayten Güleç, MD	Division of Pediatric Neurology, Department of Pediatrics, Faculty of Medicine, Erciyes University, Kayseri, Türkiye	Major role in the acquisition of data; analysis or interpretation of data
Colette Berger, MD	Department of Clinical and Functional Neurology, University Hospital of Lyon, Pierre-Bénite, France	Major role in the acquisition of data; analysis or interpretation of data
Dorothee M. Ville, MD	Department of Clinical and Functional Neurology, University Hospital of Lyon, Pierre-Bénite, France	Major role in the acquisition of data; analysis or interpretation of data
Julitta de Bellescize, MD	Department of Paediatric Clinical Epileptology, Sleep Disorders and Functional Neurology, University Hospitals of Lyon, France	Major role in the acquisition of data; analysis or interpretation of data
Sara Cabet, MD	Pediatric and fetal imaging department, Femme-Mère-Enfant Hospital, Hospices Civils de Lyon, Claude Bernard Lyon 1 University, France	Major role in the acquisition of data; analysis or interpretation of data
Antje Wonneberger, MD	Department of Neuropediatrics, Jena University Hospital, Jena, Germany	Major role in the acquisition of data; analysis or interpretation of data
Alexander Schulz, MD, PhD	MVZ Mitteldeutscher Praxisverbund Humangenetik GmbH, Johannesstr. 147, Erfurt, Germany	Major role in the acquisition of data; analysis or interpretation of data
Agusti Rodriguez-Palmero, MD, PhD	Pediatric Neurology Unit, Pediatrics Department, Hospital Universitari Germans Trias i Pujol, Universitat Autònoma de Barcelona, Spain	Major role in the acquisition of data; analysis or interpretation of data
Nicolas Chatron, MD	Department of Genetics, Hospices Civils de Lyon; NeuroMyoGene Institute, CNRS UMR 5261-INSERM U1315, Claude Bernard Lyon 1 University, France	Drafting/revision of the manuscript for content, including medical writing for content; major role in the acquisition of data
Gaetan Lesca, MD, PhD	Department of Genetics, Hospices Civils de Lyon; NeuroMyoGene Institute, CNRS UMR 5261-INSERM U1315, Claude Bernard Lyon 1 University, France	Drafting/revision of the manuscript for content, including medical writing for content; major role in the acquisition of data
Hüseyin Per, MD	Division of Pediatric Neurology, Department of Pediatrics, Faculty of Medicine, Erciyes University, Kayseri, Türkiye	Major role in the acquisition of data; analysis or interpretation of data
Himanshu Goel, MBBS, MD, DM, FRACP	Hunter Genetics, Waratah; University of Newcastle, Callaghan, Australia	Drafting/revision of the manuscript for content, including medical writing for content; analysis or interpretation of data
Janis Brown, PhD	John Hunter Children's Hospital, Australia	Drafting/revision of the manuscript for content, including medical writing for content; analysis or interpretation of data
Tanja Frey, MD	Institute of Medical Genetics, University of Zurich, Switzerland	Drafting/revision of the manuscript for content, including medical writing for content; analysis or interpretation of data
Katharina Steindl, MD	Institute of Medical Genetics, University of Zurich, Switzerland	Major role in the acquisition of data; analysis or interpretation of data
Anita Rauch, MD	Institute of Medical Genetics, University of Zurich, Switzerland; University Children's Hospital Zurich; University of Zurich Research Priority Program ITINERARE: Innovative Therapies in Rare Diseases, AdaBD: Adaptive Brain Circuits in Development and Learning, Switzerland	Major role in the acquisition of data; analysis or interpretation of data
Mariasavina Severino, MD	Neuroradiology Unit, IRCCS Giannina Gaslini Institute, Genoa, Italy	Drafting/revision of the manuscript for content, including medical writing for content; major role in the acquisition of data; analysis or interpretation of data
Henry Houlden, MD, PhD, FRCP, FRCPath, FMedSci	Department of Neuromuscular Disorders, UCL Queen Square Institute of Neurology, UCL, London, United Kingdom	Drafting/revision of the manuscript for content, including medical writing for content; major role in the acquisition of data; study concept or design
Paola Nicolaides, MB, CHB, MRCP, FRCPCH	University of Nicosia Medical School, Nicosia, Cyprus	Drafting/revision of the manuscript for content, including medical writing for content; major role in the acquisition of data
Pasquale Striano, MD, PhD	Department of Neurosciences, Rehabilitation, Ophthalmology, Genetics, Maternal and Child Health, Università Degli Studi di Genova	Drafting/revision of the manuscript for content, including medical writing for content; major role in the acquisition of data; analysis or interpretation of data
Stephanie Efthymiou, PhD	Department of Neuromuscular Disorders, UCL Queen Square Institute of Neurology, UCL, London, United Kingdom	Drafting/revision of the manuscript for content, including medical writing for content; major role in the acquisition of data; study concept or design; analysis or interpretation of data
